# Advancement of Near Infrared-II Organic Dyes in Bioimaging

**DOI:** 10.7759/cureus.47617

**Published:** 2023-10-25

**Authors:** Nidhi Sohrot, Manjusha Agrawal

**Affiliations:** 1 Obstetrics and Gynaecology, Jawaharlal Nehru Medical College, Datta Meghe Institute of Higher Education and Research, Wardha, IND

**Keywords:** near infrared 2 imaging, bioengineering, biomolecules, imaging technology, fluorescence

## Abstract

In recent decades, small organic compounds having absorption and fluorescence emission in the second near-infrared (NIR-II, 1000-1700 nm) bio-window have attracted a lot of interest. Fluorescence bioimaging may be used by researchers and surgeons to genomically focus an array of biological areas and functions. The near-infrared-II (NIR-II) dye which has fluorescent imaging, bypasses the visible imaging striking barrier, making it a valuable tool for cancer early detection and very sensitive tumor resection. It can generate sub-cellular density scanning data directly and has been applied to biological and medical detection and therapy. This paper discusses the history and current state of theranostics and biosensing uses of NIR-II tiny organic producers depending on multiple skeletons. For biological imaging, organic dyes are extensively used as markers for near-infrared (NIR) fluorescent though the issue lies in instability and hydrophobicity for bio environment which is a major restriction for its utilization. Various conjugation with the probes is also adopted in order to increase the biosensing power and efficiency and to deduct their level of cytotoxicity. Some of these combinations are discussed in the paper including supramolecule usage, combining the probes with quantum dots, and an alloy of gold selenium. NIR-II fluorescence devices are also used in combination with confocal microscopy to study the cytological interaction of proteins. Several research papers stated using cell membrane enhancement units empowered with oxazolepyridine and coumarin compounds. As the need for bioimaging increases decade by decade these cons of using organic dyes alone are getting overlapped by compounding these dyes with materials that help in better penetration, bioavailability, and reduction in areas of toxicity.

## Introduction and background

Optical imaging technology has grown in importance in biomedicine, owing to its capability to scan bio-molecules, cells, tissues, and organisms in real-time and different aspects of visualizing organisms [1]. Applied medicine with optical imaging consists of enormous priority, including the following: non-invasive and risk-free with excellent visualization capabilities, spatial resolution, fast output speed, and a reasonable cost method. Longer wavelengths have less refraction, absorbance, and a ratio of near-infrared (NIR) light (700-1700 nm) that penetrates biological tissues better than visible light (400-700 nm). Natural diagnosing types of machinery, when used and imaged under the range of wavelength that lies within visible spectra, are observed to show glitches in terms of impaired tissue visualization, reduction in resolution, deranged sensing, and contrasting potential [2-4]. Imaging using fluorescence dyes alone is now shortly used, concerns lie within its minimal tissue perfusion and inability to get washout in urine. In order to overcome this issue, the near-infrared wavelength II dyes within the range of 1000-1700 nm are now becoming the front foot in the field of imaging under biomedicine. Various conjugation of near-infrared II (NIR-II) fluoroprobes is developed to increase their bio utility some of these include polymer nanocarriers to capture NIR dyes [3].

Inorganic nanoparticles or quantum dots upconversion nanoparticles, and organic dyes are some of the particulars involved in making the NIR-II probes [4]. Inorganic nanoparticle usage comes with a wide range of disadvantages including cytotoxicity low yield and poor excretion rate. To empower these obstacles, organic dyes are adopted as a marker of NIR-II probes in bioimaging; still, these dyes are not stable in the bio environment, therefore, conjugation with carrier materials is now used [5-7]. Quantum dots labeled NIR-II probes are considered to be low in toxicity and high in terms of their performance and efficiency [8]. The troublesome task of conducting NIR-II probes along with quantum dots to synthesize a high quantum yield device considering the upper level of photoluminescence has motivated various research within bioimaging and NIR-II devices. One such research was conducted by Yang et al. [9] in which an alloy of silver gold selenide quantum dots was produced that resulted in more than double the photoluminescence with NIR-II quantum dots along with higher longevity and minimal to no toxicity [9]. Another way to improve the bio efficiency of NIR-II probes is combining them with microscopy, a confocal that uses fluorescence optics to generate images of in vivo protein interaction with proteins and their cross-linking via a dedicated beam. This collaboration has achieved greater resolution power of visualization at the extent of deeper penetration [10].

Cell membrane enhancement of fluorescence in the NIR-II probes is experimented with by Yu et al. [11]. This experiment included β-cyclodextrin supramolecular and their combination with the probes. Cell membrane units are prepared by condensation of aldol reaction substrates oxazolepyridine and coumarin. This resulted in an increment of residual absorption even on illumination for about six hours [11]. The quantum yield of the probes has now increased to more than a quarter of the previous ones. This system of addition of supramolecules is now considered the best plan to enhance the fluorescence of organic dyes [12]. The NIR-II transparency window might lead to enhanced definition, greater penetration bioimaging (ca.5-20 mm) and lower photon scattering compared to the bands of visible and NIR-I range. Small organic compounds with a high rate of urine excretion and flexible architectures that can be directly targeted to biomolecules using various conjugation methods are a better choice. Minor molecular structure changes can easily alter their optical qualities.

## Review

Search methodology

**Figure 1:**
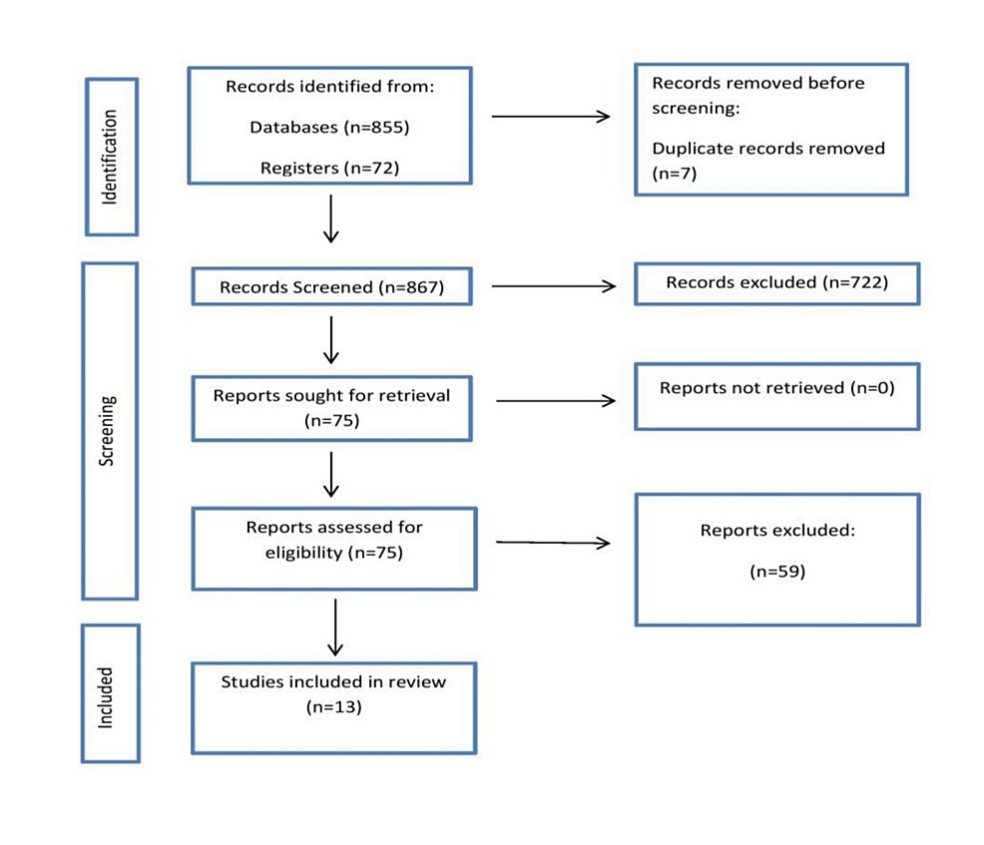
Identification of studies via databases and registers.

We undertook a comprehensive search through PubMed and CENTRAL (Cochrane Central Register of Controlled Trials) in February 2021 using keywords such as near-infrared 2 imaging, bioengineering, biomolecules, imaging technology, and fluorescence. We additionally searched for key references from bibliographies of the relevant studies. The search was updated in September 2022. One reviewer independently monitored the retrieved studies against the inclusion criteria, in the beginning, based on the title and abstract and then on full texts. Another reviewer also reviewed approximately 20% of these studies to validate the inclusion of studies (Figure 1).

Organic fluorescent dyes

In fluorescence imaging, the dyes that fall under the organic fluorescent category are still the most extensively employed luminous identifiers within the diagnosing domain. Because of their tremendous advantages in various aspects like luminescence efficiency, ease of functionalization, and a varied luminosity spectrum, organic fluorescent dyes are a choice of agents for imaging. To improve biocompatibility in vivo, organic compounds with poor water solubility must be synthesized within a hydrophilic matrix. Widely used organic fluorescent dyes include indocyanine green, C700-X, C800-X series, methylene blue, and boron dipyrromethene [13].

Indocyanine Green

The US Food and Drug Administration (FDA) has approved it as the NIR optical imaging contrast agent for clinical use. Indocyanine green (ICG ) is a near-infrared fluorescent dye with excellent absorbing properties, minimal hazard, no biological change in vivo, and quick elimination. ICG has a long history of application in the cardiovascular system, screening of liver functions, retinal and macular screening, ocular angiography, cortical angiography, and other specialized care. A recent experiment by Starosolski et al. [14] proposed that at roughly 1,100 nm, ICG produces a large number of illumination in the NIR-II window, which is controlled by ICG's molecular environment (as is the absorption curve). In vivo imaging studies revealed that NIR-II fluorophore uses ICG, with a carrier to noise level double that of the near-infrared I window. In an experiment by Bhavane et al. [15] they used NIR-II fluorescence imaging along with a dye called indocyanine green nanoparticles; investigation results revealed that the indocyanine green liposome had significant fluorescence in NIR-II window. Liposome ICG improves contrast compared to long-term indocyanine green that is not bound and allows for visualization of the hind limbs and brain arteries within four hours of injection.

Superiority Over Ultrasonography and Other Nanoparticles

When set side by side with X-ray, computed tomography in imaging vascular structures and analyzing measurements within the blood, NIR fluorescent dyes offer innocuous and robust imaging and a more excellent dynamic range of plasma flowmetry than ultrasonography. Various other nanoparticles like heavy metal compounds' potential toxicity (e.g., lead sulphide, lead selenium, mercury telluride) and limited water solvent properties have impeded usage of these metals in bioengineering. Because of their decreased toxicity, excellent bioactivity and superiority in hydrophilic, organic fluorophores are a better choice for in vivo imaging.

Applications of organic dyes in bioimaging within biomedicine

Medical Diagnosis and Testing

In the NIR-II window, deeper tissue can be visualized via fluorescence bioimaging, carrying very minimum self-fluorescence and tissue scattering. Nonetheless, detecting NIR-II fluorophores in vivo focuses on the fundamental dynamic diseased lesions or living organisms. Liu et al. report a novel Erbium-sensitized up-conversion of nanoparticles with the characteristics of both 1,530 nm excitation and 1,180 nm emissions in the NIR-II window, which showed its usage in vivo biosensors in his paper published in Angewandte Chemie International Edition in 2018. Finally, in vivo dynamic detection of inflammation has been achieved [16]. NIR-II radiometric probe is advantageous because of its significant anti-stokes dislocation, low optical background fluorescence, low absorbance and scatter in biological materials the probe can assist in detecting inflammatory reactions in vivo in live time with excellent picture quality and their refined size. It is an accurate biological imaging tool and is versatile as a perfect contrast agent. The strength of the fluorescence rises by up to 44 times, and the photostability is excellent. For example, Zhao et al. reported an approach that uses real-time bridge bonding of fine lanthanum nanoparticles in combination with glutathione with near-infrared fluorescence emission to image inflammation in vivo accurately [17]. In the inflammatory region, glutathione (GSH) mixed lanthanide nanoparticles react with reactive oxygen species (ROS) to accurately detect the location and imaging of reactive oxygen species under a window where the near-infrared range lies.

In 2019, Li et al. experimented with these fluorescence probes to image higher contrast and estimate pH. Peroxynitrite-activated NIR-II fluorescence probe was developed for hepatic injury caused due to drug-related causes detected in 2019 [18]. The up-regulation of peroxynitrite (ONNO^-^) is the primary element causing drug-related liver pathology in the model of drug-induced hepatic diseases within the incubation period, as well as the real-time repair effect of N-acetylcysteine (NAC), could be made out with this probe. When compared to the NIR first window, the NIR second window, which lies between 1000 to 1700nm of wavelength mechanics, has minor autofluorescence, a broader tissue impaling ability, a generous proportion of signal to noise with a higher difference between the position of the full spectrum of first absorption band to that of maximum emission of fluorescence, that is usually demonstrated in its wavelength (also known as Stroke Shift) [19].

Imaging of Neoplasm and Image-Assisted Surgery

With the help of fluorescence imaging to guide the removal of a particular cancer and significantly improve the prognosis, cytopenic surgery aims to enhance staging and shrink tumors. Fluorescent nanoprobes tailored with DNA and distinct polypeptides are used to assure the accuracy of cytoreduction operations in advanced carcinoma. This is only because of their excellent resistance to light and deep penetration into tissue. Under the direction of NIR-II bioimaging, metastases smaller than 1 mm might be eliminated. Zhang et al. effectively developed a biomimetic NIR-II fluorescence nanoprobe with ultra-bright fluorescence stability, similar to targeting excellent biocompatibility that can improve living tumors' scanning and image detection [20].

Imaging of Lymphatics for Diagnosis and Therapeutics of Tumor

NIR fluorescence imaging has lately been applied in the clinic for lymph tracking, operation guiding, and rapid functional imaging. It has previously been shown in laboratory animals such as mice, rats, dogs, and pigs [21]. In the study conducted in Japan by Kitai et al. which was the first use of these probes for the clinical purpose of lymphatics imaging in which eighteen breast cancer patients had 25 mg of indocyanine green (ICG) injected near the areola for visualisation of the flowing subcutaneous lymphatics and to locate the sentinel nodes [22]. Ogasawara et al. evaluated lymphatic drainage channels from numerous parts of the breast in breast tumour patients using a 25 mg dose of ICG [23]; therefore, when compared to other reagents, fluorescent dyes with a high signal-to-background (SBR) ratio and detection depth, such as ICG, provide greater sentinel lymph node detection [24].

So far, the advantages and disadvantages of NIR fluorescence imaging for non-invasive medical imaging have only recently been examined in the lymphatic system, where nuclear imaging techniques such as scintigraphy and radioimmunoscintigraphy have remained "gold-standard" imaging techniques. The potential intraoperative advantage of using an NIR active fluorescent dye over a blue dye may be the capacity to detect subterranean lymphatic systems that would be difficult to detect with direct visual inspection of the blue dye [25-27].

Drug Delivery System

The implication of organic fluorescent dyes in the drug delivery system has been widely in use recently; the efficiency of these particles is to decrease the side effects by prominently commanding the dosage of drugs and their location in the loading of drugs. This property is also helpful in increasing its remedial power [28]. On appropriate stimulus by pH, light, and biological molecules, there is drug release attainment with the help of nanoparticles. This can be achieved by their property of controlling, not invasion, high resolution with nominal damage to the tissues [29].

Biological Imaging of Vascular Tissues

The NIR-II fluorescence has many advantages and superiority over the most frequently used techniques in clinical diagnosing fields such as brightness-mode ultrasonography (USG), computed tomography (CT), and nuclear magnetic resonance (NMR) for detection and diagnosing cardiovascular and cerebrovascular diseases. NIR-II fluorescence devices already developed, delivered intravenously, and instilled within animals can scan the vasculature in vivo. In a paper by Li et al., published in 2018, it has been shown that the NIR-II area has been proven to have better resolution and penetration in biological media [30]. It uses dynamic pictures of respiratory craniocaudal motion within the liver to quantify respiratory frequencies in awake and anesthetized mice. The result demonstrated that NIR-II fluorescence could penetrate mice's skulls to a deeper level, allowing researchers to image the territory of blood vessels in the crown and the microcapillary structures. The use of NIR-II in inflammation pathology is confirmed by Wang et al.'s research article published in 2019 that stated using a 1550 nm probe to recognize the complicated lymphatic system in the inflammatory cascade [31]. A group of researchers created a nanoprobe that generated NIR-II fluorescence and was triggered by an 808 nm laser for tiny tumor identification and angiography. With the NIR-II scanning devices, researchers used visual imaging to navigate the detection of tumors, endothelial dysfunction illnesses, and angiogenic diagnostics [32]. Another level of fluorescence scanning technology, which is a type of technology that displays tumor and vascular abnormalities that can be visualized by NIR-II optical imaging with the wavelength of emission that lies in >1500 nm [33,34], can therefore be utilized to diagnose tumors early and identify tumor-related vascular features. Finally, the results showed that dual imaging of the circulatory system and malignancies was quite good.

Imaging of Brain Tissue

**Figure 2:**
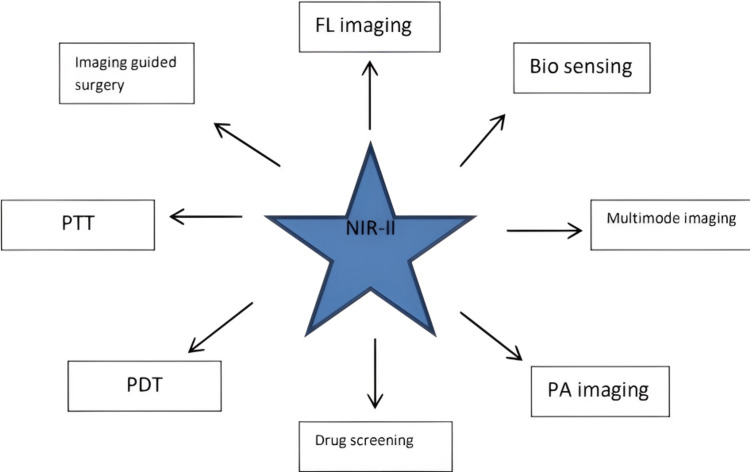
Application of organic NIR-II dyes as a medium of bioimaging. FL= Fluoroscopy; PTT= Photothermal Therapy; PDT= Photodynamic Therapy; PA= Photoacoustic Therapy; NIR-II= Near Infrared-II

The human brain controls the various sensory and motor functions of the body with afferent and efferent innervations at different levels. The diseases affecting the brain do not always carry a good prognosis due to delays in diagnosing and the inability to image the micro-damaged areas. With advancements in bioimaging with nanofluorescent particles, medical science has advanced in diagnosing lesions involving the brain parenchyma over time, especially using NIR-II particles [35]. In a comparison of MRI, the rate of functional MRI (fMRI) detects the activity inside the brain neural pathway only via quantifying the variations in the flow of blood within the parenchymal tissue [36]; this mechanism of detection of lesions corresponds to the relation between the flow of blood and the neuronal activity and polarization of action potential within the brain. The target molecules of these fluorescent probes are proteins, nucleic acids, glycoproteins, and lipids that are endogenously synthesized and therefore, are easily detected with the help of a suitable antibody. Tagging and labeling of these biomolecules are made achievable by using green fluorescent protein within a living cell of the organism [37]. NIR-II dyes as a means of bioimaging are used in various aspects of biomedicine. Figure 2 shows some of these applications of organic NIR-II dyes in bioimaging.

Widely used NIR-II organic molecules in bioimaging

Organic molecule dyes along with benzobisthiadiazole (BBTD) acceptors were used by Wang et al. as they synthesized structured organic fluorophores having emission ranges in the NIR-II window [38]. They used benzobisthiadiazole as an acceptor of electrons with some donors and benzene was used as a medium to augment the transfer of charge within the molecule. Polymethine cyanine dyes such as polymethinefluorophores, employed without a BBTD core, are among the most important classes. Many well-known NIR-II emission molecules such as IR-1040, IR-1048, and IR-1061, were produced by scientists, and some of them are now commercially accessible compounds [38]. Other Dyes including methylene blue, is a therapeutically licensed NIR dye with a low range of wavelength emission of nearly equal to 686 nm that is extensively used while undergoing imaging during operative procedures for therapy of methemoglobinemia [39].

**Figure 3:**
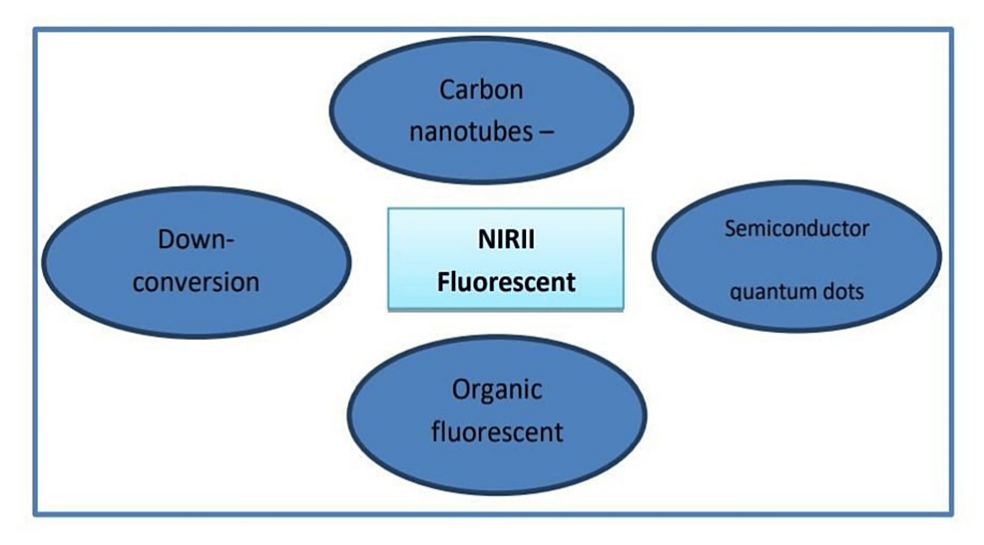
Widely used NIR-II fluorescent dyes. NIR-II= Nearinfra Red-II

The C700-X and C800-X class of contrast agents have been described as cartilage-specific. Boron-dipyrromethene dyes are intensively labelled as detectors or indicators and labelling mediums, forming an essential basis in molecular imaging and the administration of drugs. The benzo[a]phenoxazine family, which has intrinsically excellent optical properties and variable framework, is vital in NIR fluorescence and probes dyes. These compounds have demonstrated notable roles in the medicinal domain, such as tumor growth suppressors and activity against tuberculosis and fungal diseases [40,41]. Nile red and the Nile blue pH and polarity indicators with strong quantum yields and solvent-dependent optical characteristics [42]. CDr20 (red 20 cell designation of the compound) is adapted to know the activity of an enzyme called uridine diphosphate (UDP) glucuronosyl transferase. CDg16 (a marker of compound green 16) for the vector of solute 18b1 is progressed in biosensing areas because these organic compounds are small in size and, therefore, do not lead to any moderation at the gene level. When introduced within the soft brain tissue, these dyes function by crossing the blood-brain barrier, thereby leaving their biochemical positioning intact within the blood vasculature. Figure 3 shows some commonly used NIR-II dyes in different domains of bioimaging.

Results

Compared to standard near infrared-I (NIR-I) imaging and other medical imaging techniques such as ultrasonography, computed tomography (CT) scans, and magnetic resonance imaging (MRI) NIR-II technology of bioimaging, along with providing greater image precision, also reduces background disturbances like sudden fluorescence and scattering of photons within a tissue [43]. NIR-II bioimage probing detects deep tissue characteristics at centimeter depths and achieves even micron resolution at millimeters, much beyond NIR-I fluorescence imaging capabilities. All these modalities of investigations used clinically for determining and finding the disease causation are deprived of sensitivity and specificity and carry possible radiological-related threats [44,45]. NIR-II imaging contrast agents have advanced in diagnosing cancer via imaging and scanning, medical detection of diseases, and imaging at the vascular level. Moreover, while visualizing organs such as the brain in the visible waveband, the resolution is little when visualizing with NIR-II probes because of barrier properties of various soft tissue linings such as skin, muscle, fat, and bone, making the invasion difficult. While using NIR-II dyes, these obstacles are being overcome in non-invasive bioimaging [46,47].

## Conclusions

We expect to see a lot more brilliant commercially available NIR-II luminous probes that are therapeutically available when surgeons need to evaluate cancer edges and map specific anatomical characteristics in therapeutic trials. Because small organic NIR-II fluorophores have various optical properties, advantageous bioavailability, low toxicity, and physical properties, as well as chemical properties, can be readily changeable by doing modifications to their structure, they hold a lot of promise in the field of modern surgery, especially in the area of focused surgery guiding, which has the potential to transform. Per recent discoveries, the terminal ends of the NIR-I dyes indocyanine green (ICG) and methylene blue (MB) fluorescence spectra can be lengthened to the NIR-II band, allowing for the clinical implementation of NIR-II imaging technology for clinical surgical localization.

With varieties of leading advantages of NIR-II fluoroprobes, there are also some disadvantages in their incapacity to withstand water and get disrupted via reactive oxygen species. Also, they have low quantum outcomes in terms of yields. Therefore, work needs to be done to reduce the bandwidth of these probes' chromatic wavelength, which would conserve the power to remain stable in liquid solutions. Another disadvantage lies in their property to cause possible harm to the human body because these nanoprobes are toxic to human tissue without having an appropriate clearing mechanism from the body and their metabolism within the human tissues. In the last design, the advancement and functioning of the NIR-II imaging are essential. In a low field of visualization, lesser firmness and meager power of depth invasion restrict their use in optical imaging. Photoacoustic imaging tends to amplify the depth of scanning and image-guiding diagnosis in the NIR-II window. It is critical to create novel multifunctional and multiscale imaging methods, as well as multi-contrast agents, for accurate tumor detection and surgical routing. NIR dye imaging, particularly NIR-II derived imaging is still in its early stages, necessitating more high-performance compounds with customizable emission spectra and high electron efficiencies being developed. To fully fulfill a chance of NIR imaging, new NIR-generating compounds with greater intrinsic efficiencies and groups of functions for crosslinking must be developed and regulated for human use.
